# Simultaneous gains in grain yield and nitrogen efficiency over 70 years of maize genetic improvement

**DOI:** 10.1038/s41598-019-45485-5

**Published:** 2019-06-24

**Authors:** Sarah M. Mueller, Carlos D. Messina, Tony J. Vyn

**Affiliations:** 1 CiBO Technologies, St. Louis, MO 63102 USA; 2 Corteva AgriScience, Johnston, IA 50310 USA; 3 Purdue University, Agronomy Department, West Lafayette, IN 47907-2054 USA

**Keywords:** Plant breeding, Plant physiology

## Abstract

The competing demands of increasing grain yields to feed a growing population and decreasing nitrogen (N) fertilizer use and loss to the environment poses a grand challenge to farmers and society, and necessitates achieving improved N use efficiency (NUE) in cereal crops. Although selection for increased yield in maize has improved NUE over time, the present understanding of the physiological determinants of NUE and its key components hampers the design of more effective breeding strategies conducive to accelerating genetic gain for this trait. We show that maize NUE gains have been supported by more efficient allocation of N among plant organs during the grain filling period. Comparing seven maize hybrids commercialized between 1946 and 2015 from a single seed company in multiple N fertilizer treatments, we demonstrate that modern hybrids produced more grain per unit of accumulated N by more efficiently remobilizing N stored in stems than in leaves to support kernel growth. Increases in N fertilizer recovery and N harvest index at maturity were mirrored by a steady decrease in stem N allocation in this era study. These insights can inform future breeding strategies for continued NUE gains through improved conversion efficiency of accumulated plant N into grain yield.

## Introduction

Nitrogen fertilizers created by the Haber-Bosch process have been credited with feeding 48% of the world’s population^1^, but the negative effects of added reactive N to the environment are a global concern^2–5^. The competing demands of increasing grain yields to feed a growing population and decreasing N fertilizer use and loss to the environment poses a grand challenge to farmers and society, and necessitates improved N use efficiency (NUE) in cereal crops^6–10^. Nitrogen use efficiency is defined as the amount of grain produced per unit of N accumulated above what is provided by soil N mineralization. The selection for increased yield in maize has resulted in higher NUE over time^11–13^, but an understanding of the physiological determinants of NUE is lacking, and needed, to design breeding strategies conducive to increasing genetic gain for this trait. Era studies comparing maize hybrids commercially released over multiple decades, in the presence of intentional abiotic stress factors, are especially valuable for discerning physiological determinants of yield gains^14^.

The United States currently produces 36% of the world’s maize supply^15^. This high production has been possible because maize farmers in the U.S. began utilizing hybrids when they were introduced in the 1930’s, and over time have adopted progressively superior-yielding genetics. Rapid adoption of N fertilizers began in the 1960’s concurrently with the introduction of higher yielding single-cross hybrids^14^. Breeding selection has almost exclusively focused on increasing grain yield and agronomic attributes^16^, resulting in a 4-fold increase in maize yields from 1930 to 2014^17^. Most experiments find that grain yield has increased linearly with hybrid improvement, and that the rate of maize yield increase is steeper under optimal conditions compared to stress environments including low N^11,13,18^ or drought^19,20^. Breeding for higher yields has also increased total biomass and N accumulation at maturity, extended duration of green leaf area, improved capture of photosynthetically active radiation, and decreased grain N concentration, among other changes^11,13,14,18,21^. While some of these physiological changes may be conducive to higher NUE, a systematic analysis of its determinants is lacking.

**Figure 1:**
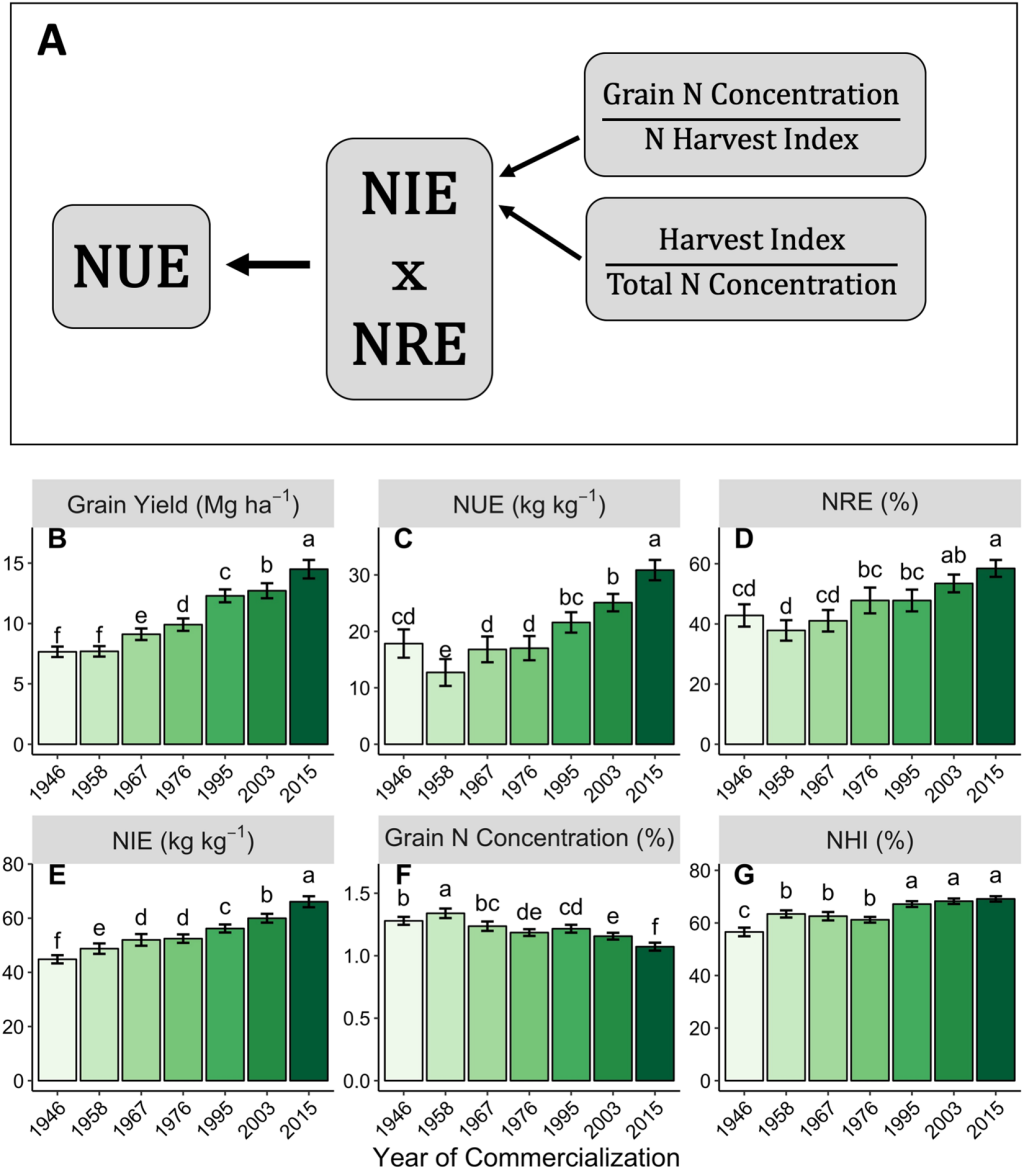
Applicable NUE component changes in maize hybrids over time. Schematic of the relationship between NUE, NIE, and NRE (**A**) and effects of maize hybrid YOC on grain yield (**B**), NUE (**C**), NRE (**D**), NIE (**E**), grain N concentration (**F**), and NHI (**G**). Bars represent standard error. Means denoted with different letters are significantly different from each other at p < 0.05. All means are presented as the average of two years and five N treatments.

A physiological framework for the analysis of NUE (Fig. 1A) considers two components: how much N is taken up by the crop (N recovery efficiency, NRE) and how efficiently N is transformed into grain yield once it has been accumulated in the crop (N internal efficiency, NIE). NIE is further broken down into the ratio between:

- N content in the grain expressed as percent of the grain mass (grain N concentration) and the nitrogen harvest index (NHI), which is the fraction of the total plant N at maturity that is present in the grain^22^
- the ratio between grain mass and total plant mass (harvest index, HI) and the N present in the plant at maturity expressed as percent of total dry matter (total N concentration)^23^

Nitrogen internal efficiency is genetically controlled and synthesis analyses across diverse environments, managements, and genotypes, have estimated that about 56 kg of grain are produced for every kg of above-ground plant N uptake^23,24^. Nitrogen recovery efficiency is typically below 50%, meaning that much of N fertilizer applied is not used by the recipient crop. Nitrogen fertilizers not taken up by the crop, or retained in soil, are lost to the environment^4,6,23^. Improvements in NRE through agronomic management has been a research focus for several decades and has resulted in best management practices for N fertilizer including optimized fertilizer source and N application timing^22,24^. In contrast, research seeking to identify opportunities for genetic improvement in NRE and NIE is only recent^11,12,25^. Understanding how NUE and its components have changed after decades of maize breeding provides a first assessment into the opportunities for intentional breeding-driven gains in NUE. Several studies have found that NUE has increased with hybrid improvement^11–13,26^. However, what has not been adequately demonstrated is the source of hybrid improvement in NUE.

As pointed out in reviews by Egli (2015)^17^ and Duvick (2005a)^14^, N fertilizer rates for maize production in the United States have plateaued since the 1980’s. However, over this same period, grain yields have continued to increase. In order to best evaluate changes in NUE, a 0 N control is needed to account for soil N accumulation and response to N fertilizer. Two experiments that have included 0 N treatments, Haegele *et al*.^11^ and Woli *et al*.^26^, found contrasting results for NUE change over time with the former noting an increase of 0.16 kg kg N^−1^ year^−1^ while the latter found no trend in NUE. The lack of difference in NUE with YOC reported by Woli *et al*.^26^ may have been partially due to the different plant populations used for each hybrid. In the absence of a 0 N control, partial factor productivity can serve as a substitute for understanding the ability of hybrids to produce grain yield per unit of N applied. Chen and Vyn (2017)^12^ and DeBruin *et al*.^13^ both reported a positive relationship between partial factor productivity and YOC. Furthermore, the role of NIE has had mixed results showing both improvement with YOC^11^ and no response. To address the knowledge gap of source of hybrid improvement in NUE over time, the objective of this study was to evaluate the mechanisms of NUE gains, through the assement of both NIE and NRE, over the past 70 years of commercial hybrid selection.

## Methods

### Experimental design and site description

The methods of this experiment are discussed in-depth in Mueller and Vyn (2018)^27^. In brief, a two-year experiment was carried out at the Purdue Agriculture Center for Research and Education in West Lafayette, Indiana (40.471, −86.992) in 2016 and 2017. This site consists of silty clay loam soil (fine-sily, mixed, mesic Typic Haplaquolls). The site was rainfed and managed in a maize-soybean rotation. A split-plot design was used with N treatment as the main plot and hybrid as the sub-plot with three replications. Nitrogen treatments included a 0 N control and four treatments which all received a total of 220 kg N ha^−1^ but varied in timing of N application. Non-0N treatments included the following where the number before the underscore indicates the rate applied at V4 and the number after the underscore represents the rate applied at R1: *22*0*_0, 55_165, 165_55, and 0_220*. These N treatments were intended to create a range of N stress at R1. Seven hybrids, representing a subset of the Pioneer ERA hybrids^28^ and a more recent hybrid were used in this experiment. The hybrids ranged in year of commercialization (YOC) from 1946–2015. Hybrid names and YOC are listed in Table 1. These ERA hybrids were recommended for this study by Pioneer scientists because of their popularity and wide use during their commercial lifespan. Hybrids ranged in crop relative maturity from 111 to 114 days and the established plant population was 78,500 plants ha^−1^. Although plant populations changed during the decades represented by these hybrids, previous era studies have also utilized a single “modern-era” plant population^11,19,20^. Several authors have attempted to separate the confounding effect of increasing plant density relative to genetic contributions to grain yield with conflicting results. While some researchers comparing hybrids at multiple plant densities do find that modern hybrids out-perform older hybrids at higher densities^13,29–31^, others find no YOC by plant density interaction^32–34^. All hybrids in this experiment were similar in silking date and the mean anthesis-silking interval did not exceed 2.1 days for any hybrid (Table 1).

**Table 1:** Description of hybrid flowering, yield components, and N remobilization.

YOC	Hybrid	R1 Days	ASI	KN	KW	% Leaf Remob	% Stem Remob
1946	352HYB	65.0 (±0.5) b	1.9 (±0.3) a	388 (±20) d	223 (±6.0) d	53.5 (±2.3) b	31.6 (±4.6) d
1958	354 A	65.0 (±0.4) b	2.1 (±0.3) a	421 (±16) c	221 (±3.7) d	60.6 (±2.0) a	44.2 (±5.2) bc
1967	3390	66.0 (±0.5) a	0.8 (±0.2) bc	453 (±18) b	219 (±4.7) de	57.9 (±2.4) ab	48.7 (±3.6) b
1976	3382	66.0 (±0.6) a	0.5 (±0.3) cd	507 (±20) a	211 (±3.0) e	47.7 (±2.5) c	36.8 (±4.0) c
1995	3335	63.7 (±0.6) c	0.1 (±0.3) d	465 (±16) b	265 (±5.3) b	57.3 (±2.5) ab	41.7 (±5.3) bc
2003	34N42	62.7 (±0.5) d	1.4 (±0.2) ab	524 (±16) a	252 (±5.2) c	53.8 (±3.0) b	49.8 (±4.7) ab
2015	P1311	64.8 (±0.4) b	−0.1 (±0.2) d	494 (±17) a	296 (±6.9) a	55.0 (±3.3) ab	58.2 (±3.0) a

Year of release (YOC), hybrid name, days from planting until 50% silking (days, R1 Days), anthesis-silking interval (days, ASI), kernel number as an average of 10 ears (kernels ear^−1^, KN), kernel weight (mg kernel^−1^, KW), percent of stem and leaf N present at R1 remobilized by R6 (percent, % Leaf Remob, % Stem Remob). All means are presented as the average of two years and five N treatments. Values in parentheses represent standard error.

The experiments were planted on 20 May 2016 and 18 May 2017 in plots 3.05 meters (4 rows) wide and 17 meters long. No starter fertilizer was applied at planting, but an in-furrow insecticide [Tefluthrin, (2,3,5,6-tetrafkuro-4-methylphenyl) methyl-(1a,3a)-(Z)-3-(2 chloro-3,3,3-trifluror-1-propenyl)-2,2- dimethylcyclopropanecarboxylate] was used at planting to protect all hybrids against corn rootworm (*Diabrotica virgifera virgifera*). Nitrogen fertilizer applications at V4 were applied via coulter-injected 28% urea ammonium nitrate (UAN). At R1 UAN was hand-applied between rows in surface-bands. Temperature and precipitation were non-yield limiting in both years. Detailed weather data, as well as soil fertility information, is provided in Mueller and Vyn (2018)^27^.

### Plant measurements and nitrogen use efficiency

Whole-plant biomass samples were collected at flowering (R1) and at physiological maturity (R6). At each of these sampling times, 10 consecutive plants were removed from the field and partitioned into stems (including tassels), leaves (including husks), and ears. At R6 ears were further divided into cobs and kernels. All biomass sampling zones were pre-selected shortly after seedling emergence. All biomass samples were dried to a constant weight, ground to 1 mm, and analyzed for N concentration using the combustion method by Pioneer (Johnston, IA). Grain yield, kernel number per ear, and kernel weight were determined from the 10 plants harvested at R6.

Remobilization of N content and dry matter from both leaves and stems were calculated as the differences between R1 and R6. We use remobilization to refer only to the N changes in the vegetative organs (i.e. leaves and stems).

Nitrogen use efficiency (NUE), N internal efficiency (NIE), and N recovery efficiency (NRE) were calculated as:

$$NUE=\frac{G{Y}_{fert}-\,G{Y}_{0N}}{N\,rate}$$

$$NIE=\frac{GY}{R6Nc}$$

$$NRE=\frac{R6N{c}_{fert}-\,R6N{c}_{0N}}{N\,rate}$$

Where GY_fert_ and GY_0N_ refer to grain dry matter (0% moisture) under the fertilized and unfertilized treatments, respectively, and R6Nc_fert_ and R6Nc_0N_ refer to the total R6 N content (kg N ha^−1^) in the fertilized and unfertilized treatments, respectively.

### Statistical analysis

Statistical analysis was conducted using SAS 9.3^35^. For the majority of measured variables, *F*-tests based on the mean square between the two years (2016 and 2017) had Pr (*F* > *F*_*0*_) > 0.01^36^ indicating homogeneity of variance between years. For this reason, the two years were pooled together and the means presented are the average of both years. Analysis of variance was conducted using PROC MIXED and means separation was determined using the LSMEANS statement in SAS. The N treatment (whole plot) and hybrid (sub-plot) were considered fixed effects. Block was treated as nested within year (block(year)) and year, block(year) and year × block(year) × N treatment were considered random effects. Standard error calculations were derived from three replications, five N treatments, and two years.

Rate of gain was calculated using the lm function in RStudio^37^ with YOC as the explanatory variable and mean (including all five N treatments) grain yield, NUE, NRE, NIE, grain N concentration, NHI, HI, stover N content, stover dry weight, total N concentration, total N content, and total dry weight as the response variables^28^.

All data are represented as the mean of all N treatements, including the 0N control plots.The interaction of YOC × N treatment was rarely significant (p < 0.05) and, therefore, discussion will be based on the average of all N treatments unless otherwise specified.

## Results and Discussion

### Grain yield improved linearly with hybrid improvement

Over the 70 years of genetic improvement in the U.S. Corn Belt represented by the seven hybrids evaluated in this experiment, grain yield increased at a rate 0.10 Mg ha^−1^ year^−1^, resulting in an 89% increase from 1946 to 2015 when averaged across all N fertilizer treatments (Fig. 1B, Table 2) There was no evidence that modern hybrids responded differently to the non-0 N treatments and, in all cases, grain yield increased linearly with YOC. The rate of yield gain was much greater when 220 kg N ha^−1^ was applied (0.12 Mg ha^−1^ year^−1^, average of four treatments) compared to when there was no fertilizer added (0.05 Mg ha^−1^ year^−1^) (data not shown).

**Table 2:** Rate of gain for selected hybrid traits.

Variable	Slope	StdEr	*R* ^2^	p-value
Grain Yield (Mg ha^−1^)	0.10	0.007	0.98	<0.001
NUE (kg kg^−1^)	0.21	0.052	0.77	0.009
NRE (%)	0.26	0.056	0.81	0.006
NIE (kg kg^−1^)	0.28	0.026	0.96	<0.001
Grain N (%)	−0.003	0.001	0.77	0.010
NHI (%)	0.16	0.031	0.84	0.003
HI (%)	0.19	0.015	0.97	<0.001
Stover N (kg ha^−1^)	−0.08	0.056	0.28	0.221
Stover DW (Mg ha^−1^)	−0.0004	0.007	0.0007	0.956
Total N (%)	−0.0007	0.0003	0.60	0.042
Total N (kg ha^−1^)	0.55	0.060	0.94	<0.001
Total DW (Mg ha^−1^)	0.06	0.008	0.94	<0.001

Rate of gain for selected traits as calculated using linear regression with year of commercialization as the explanatory variable and the trait of interest as the response variable. Calculations were conducted using the mean of two years and five N treatments. Varibles are grain yield (Mg ha^−1^), N use efficiency (NUE, kg kg^−1^), N recovery efficiency (NRE, %), N internal efficiency (NIE, kg kg^−1^), grain N concentration (Grain N, %), N harvest index (NHI, %), harvest index (HI, %), stover N content (Stover N, kg ha^−1^), stover dry weight (Stover DW, Mg ha^−1^), total N concentration (Total N, %), total N content (Total N, kg ha^−1^), total dry weight (Total DW, Mg ha^−1^).

The increase in grain yield (averaged over all N treatments) was driven by a combination of both higher kernel number per ear and kernel weight (Table 1). The increase in kernel number primarily occurred between 1946 and 1976 while the gains in kernel weight were most notable from 1976 until 2015.

The rate of yield gain in this experiment was similar to previous era studies. The average increase in the non-0N treatments of 0.12 Mg ha^−1^ year^−1^ is similar to the gains of 0.14 Mg ha^−1^ year^−1^ reported by DeBruin *et al*.^13^ and 0.09 Mg ha^−1^ year^−1^ found by Haegele *et al*.^11^ under similar plant densities (79,000 and 80,000 plants ha^−1^, respectively) and non-limiting N rates. Our finding of a yield increase of 0.05 Mg ha^−1^ year^−1^ when no N was applied was also similar to the 0 N^11^ or low N^13^ gains reported by those authors of 0.06 and 0.06 Mg ha^−1^ year^−1^, respectively.

### Nitrogen use efficiency increased over time

**Figure 2:**
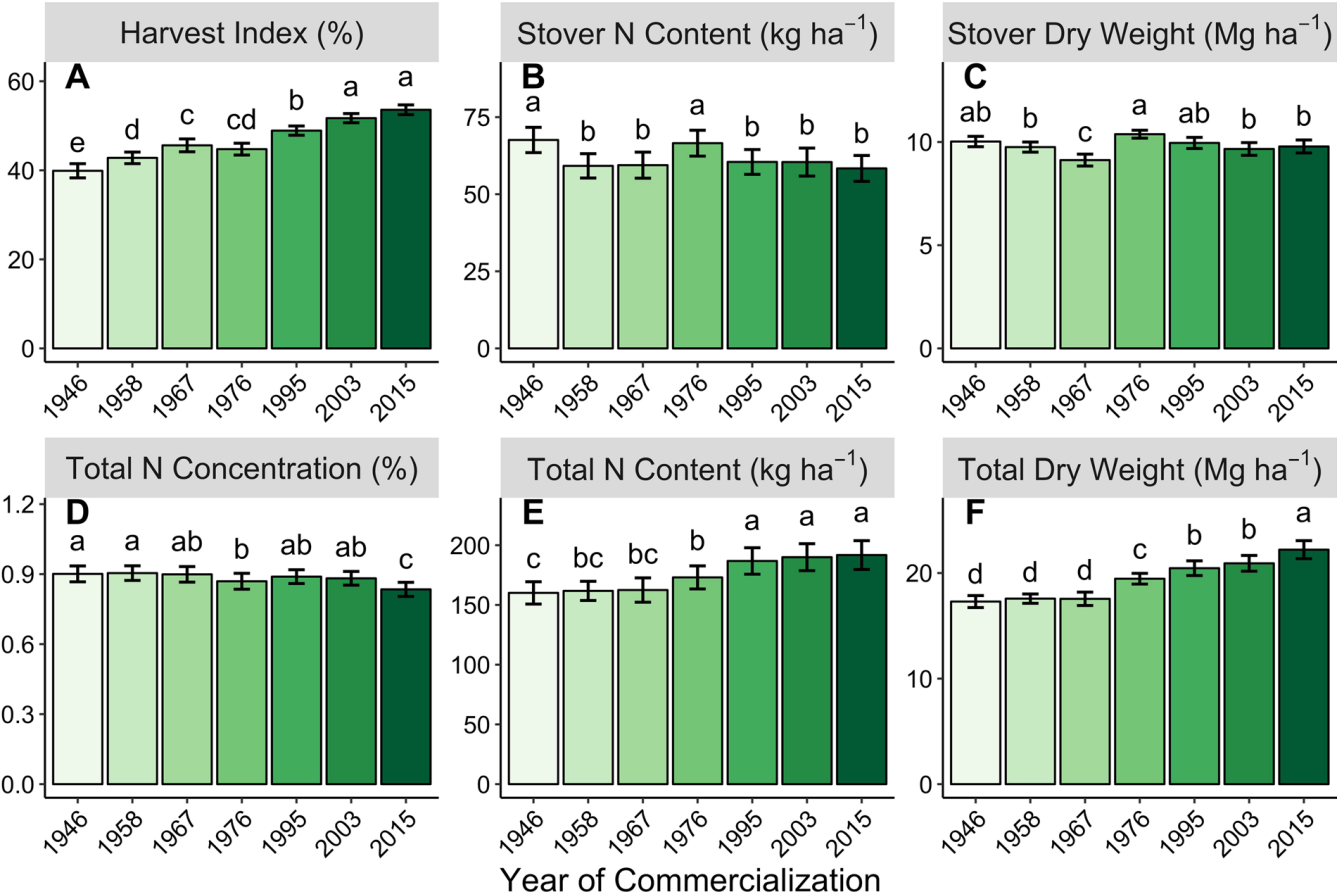
Hybrid improvement influence on end-of-season metrics. The effect of maize hybrid YOC on HI (**A**), stover N content (**B**), stover dry weight (**C**), total N concentration (**D**), total N content (**E**), and total dry matter (**F**). Bars represent standard error. Means denoted with different letters are significantly different from each other at p < 0.05. All means are presented as the average of two years and five N treatments.

In parallel with grain yield, NUE increased at a rate of 0.21 kg ha^−1^ year^−1^ (Table 2), resulting in a overall NRE increase of 73% (Fig. 1C) across the evaluated hybrids in this study. This increase was due to improvements in both NRE and NIE (Fig. 1D, E). NRE made rapid gains of 0.26 percent year^−1^ (Table 2) and this was mirrored by the strong increase in total plant N uptake at R6, which increased from 160 kg ha^−1^ in 1946 to 192 kg ha^−1^ in 2015 (Fig. 2E).

In addition to the rapid gain in NRE, modern hybrids produced more grain yield per unit of N uptake (NIE, Fig. 1E). Nitrogen internal efficiency increased nearly 50% between 1946 and 2015 (average of all N treatments), improving at a rate of 0.28 kg kg^−1^ year^−1^. This increase in NIE occurred in conjunction with a reduction in grain N concentration (numerator in NIE calculation, Fig. 1A), from 1.34 (1958) to 1.07% (2015) (Fig. 1F).

The reduction in grain protein concentration has often been implicated as having a role in improved grain yields^28,38^. However, the simultaneous increase in NHI (Fig. 1G) shows grain yields increased faster than the decrease in grain N concentration. Harvest index also increased with YOC (Fig. 2A). The change in both of these indices (NHI and HI) was driven entirely by increased grain N and grain dry matter accumulation because there was no change in stover (stems and leaves) N content (p = 0.221) or dry matter (p = 0.956) over the same period (Table 2, Fig. 2B, C). In contrast, but consistent with prior studies^13,24,25^, total N concentration at maturity decreased with YOC from 0.90 to 0.83% (p = 0.042, Fig. 2D) indicating that the increase in total N content was less than the increase in total dry matter (Fig. 2E, F). These changes suggest that improved maize grain yield has not simply been due to greater total biomass accumulation, but rather to a shift in the proportional allocation of accumulated N within the plant to the grain and away from the stover in modern maize hybrids.

### Leaf versus stem contributions to nitrogen efficiency changes over time

**Figure 3:**
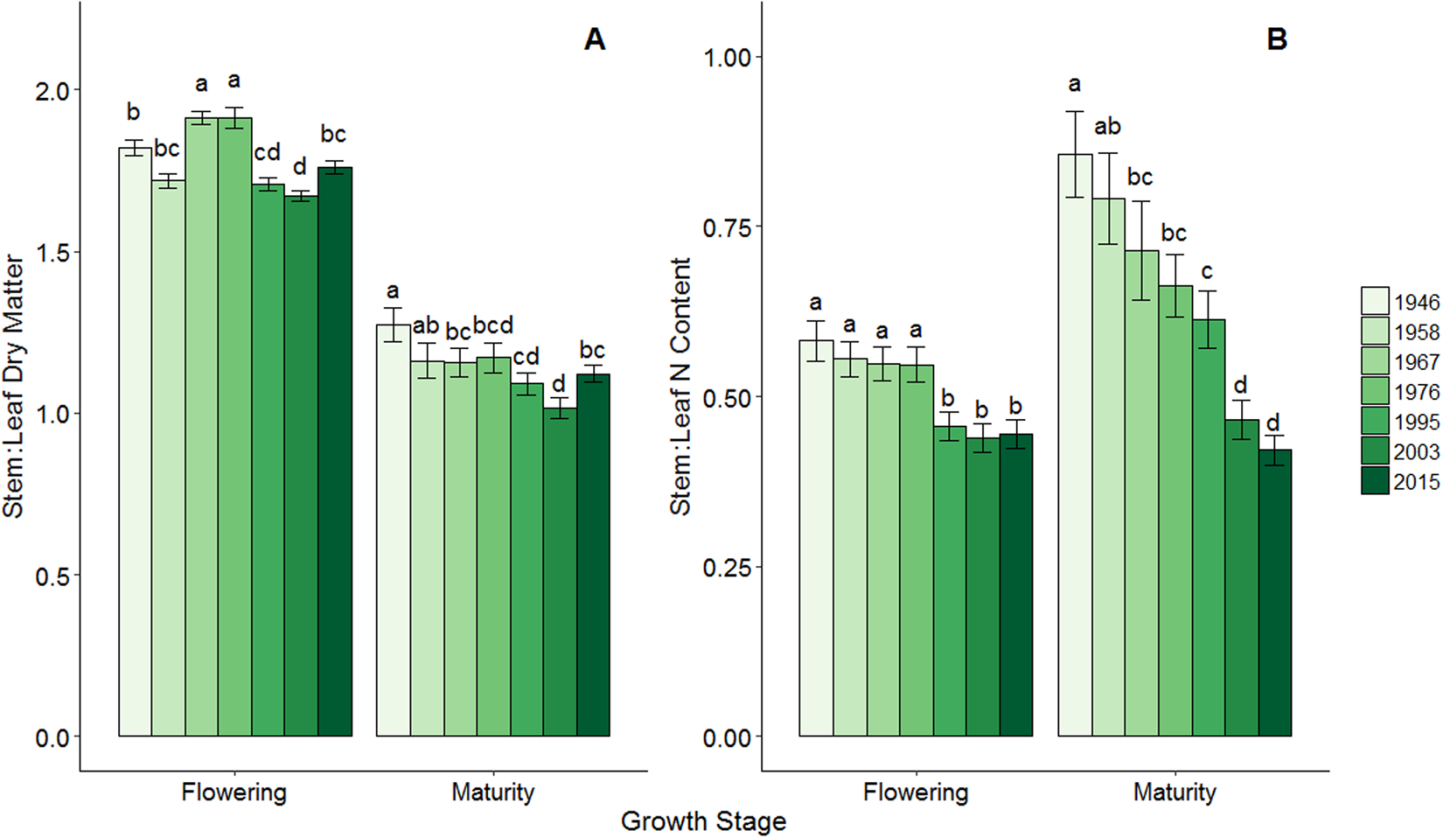
Hybrid effects on stem to leaf biomass and N ratios. The effect of hybrid YOC on the stem to leaf ratio for dry matter (**A**) and N content (**B**) at the growth stages of flowering and maturity. Bars represent standard error. Means denoted with different letters are significantly different from each other at p < 0.05. All means are presented as the average of two years and five N treatments.

Because of the different biological function of leaves and stems for yield determination, an observed increase in N allocation to the grain could be explained by changes in N allocation between these two plant organs in response to selection for yield. While leaf N determines rates of photosynthesis that support both vegetative and reproductive growth^38^, stem N is primarily structural and serves as transient storage for N accumulated in excess of that required to support plant growth^39^. The dynamic relationship between the stems and leaves during the growing season can be visualized through the stem to leaf ratio (S:L) of dry matter and N content (Fig. 3).

While experimental results show only small changes in stover dry matter or N content over the 70 years of hybrid improvement (Fig. 2B, C), major shifts occurred in S:L ratios for both dry matter and N content (Fig. 3). At flowering, stem dry matter was nearly twice that of leaf dry matter, but there was little pattern with YOC (Fig. 3A). This is in contrast with Chen *et al*. (2015a)^40^ who found higher S:L DM at R1 in Dekalb hybrids released in 1967 compared to 2005 (2.5 and 2.3, respectively). In the present experiment, by R6, S:L DM generally decreased with YOC; however, the change with hybrid improvement was much starker for S:L N content. At flowering, S:L N content was greater in hybrids released prior to 1976 (Fig. 3B). However, these differences were small compared to the prominent hybrid gradient at maturity when 1946 realized a S:L N content double that of 2015 (0.8 compared to 0.4, Fig. 3B). This demonstrates the change among hybrids in the apparent remobilization during grain filling of N content present at flowering in the stems versus the leaves. To provide the N needed for the growing kernels, maize depends on a combination of N remobilized from the stems and leaves, as well as continued N uptake from the soil during grain fill. Modern hybrids remobilized a significantly higher percentage of their stem N content at flowering (58.2% compared to 31.6% for 2015 and 1946, respectively, p < 0.001, Table 1). Meanwhile, there was no pattern between YOC and the percentage of leaf N present at flowering that was remobilized, as apparent leaf N remobilization ranged from 47.7% (1976) to 60.6% (1958) (p < 0.004). These data, except *2015*, agree with previous research showing that most of the total remobilized N originates from the leaves of maize hybrids compared to the stems^41–43^.

The greater stem N remobilization in modern hybrids led to genotypic differences in N allocation among the plant organs at maturity (Table 3) and allowed for improved NIE. As previously discussed, total dry matter at maturity increased in modern hybrids due to the greater grain dry weight while stover dry matter and N content remained unchanged (Fig. 2, Table 2). However, there were large differences in N allocation among the plant organs. As shown by the NHI results (Fig. 1G), the proportional allocation of N content to the grain increased steadily with YOC at a rate of 0.16 percent year^−1^ (Table 2). The increase in NHI was mirrored by a steady decrease in stem N allocation at R6 (Table 3) falling significantly from 17.2% in 1946 to 7.3% in 2015 (p < 0.001). There was little difference in cob N content among hybrids (representing 3.0–5.9% of the total N content, p < 0.001, Table 3) and the variation in leaf N allocation was also minimal (range of 17.2–20.9% of total N content, p < 0.001, Table 3). This analysis suggests that the improved NIE in modern hybrids can be attributed to more efficient remobilization of stem N content to the grain to support kernel growth while the proportional leaf N allocation remained relatively unchanged.

**Table 3:** Proportional N allocation among plant organs at physiological maturity.

YOC	%Grain	%Cob	%Leaf	%Stem
1946	56.6 (±1.6) c	5.9 (±0.3) a	20.3 (±0.8) ab	17.2 (±1.3) a
1958	63.4 (±1.4) b	4.8 (±0.3) ab	17.5 (±0.5) d	14.3 (±1.2) b
1967	62.6 (±1.6) b	4.8 (±0.4) ab	19.3 (±0.7) abc	13.3 (±1.2) b
1976	61.2 (±1.1) b	4.4 (±0.3) b	20.9 (±0.6) a	13.5 (±0.8) b
1995	67.2 (±1.1) a	4.8 (±0.3) ab	17.2 (±0.6) d	10.8 (±0.7) c
2003	68.3 (±1.0) a	3.0 (±0.4) c	19.5 (±0.6) bc	9.2 (±0.6) cd
2015	69.1 (±1.0) a	4.5 (±0.5) ab	19.1 (±0.8) bc	7.3 (±0.4) d

Percent of total N at physiological maturity present in the grain, cob, leaf, and stem. All means are presented as the average of two years and five N treatments. Values in parentheses represent standard error. Means denoted with different letters are significantly different from each other at p < 0.05﻿.

In conclusion, this research addressed an important aspect of the quandary for producers and society alike of simultaneous imperatives to increase grain yields and decrease N fertilizer use and environmental losses. We have shown that modern hybrids are meeting this challenge as genetic improvement in the U.S. over the past 70 years has resulted in both a 89% increase in grain yields and a 73% increase in NUE. Modern hybrids are better at accumulating a greater percent of applied N fertilizer (NRE), which limits environmentally damaging field nutrient losses while more efficiently producing grain yield per unit of accumulated N (NIE). Although previous research has suggested that maize grain yield and NUE gains over time have arisen primarily from greater total N accumulation and dilution of grain N concentration, we provide novel evidence that increased stem N remobilization, and retention of leaf N during reproductive growth, played key roles in achieving NIE gains. Historically, green leaf area development, architecture, and duration have received the focus in maize yield improvement programs, but results from this study suggest there are additional prospects for genetic improvement in NIE. Understanding the physiological underpinnings of NIE such as the dynamics and form of stem N storage and remobilization, and transporter activity during the grain filling period, are warranted to help design breeding strategies to select genotypes with increased NRE and NIE.

## References

[1] Erisman JW, Sutton MA, Galloway J, Klimont Z, Winiwarter W (2008). How a century of ammonia synthesis changed the world. Nat. Geosci..

[2] Sutton MA (2011). Too much of a good thing. Nature.

[3] Bodirsky, B. L. *et al*. Reactive nitrogen requirements to feed the world in 2050 and potential to mitigate nitrogen pollution. *Nat. Commun*. **5** (2014). 10.1038/ncomms4858 24819889

[4] Lassaletta L, Billen G, Grizzetti B, Anglade J, Garnier J (2014). 50 year trends in nitrogen use efficiency of world cropping systems: The relationship between yield and nitrogen input to cropland. Environ. Res. Lett..

[5] Keeler, B. L. *et al*. The social costs of nitrogen. *Sci. Adv*. **2** (2016). 10.1126/sciadv.1600219 PMC5052012 27713926

[6] Cassman KG, Dobermann AR, Walters DT (2002). Agroecosystems, nitrogen-use efficiency, and nitrogen management. Ambio.

[7] Tilman D, Cassman KG, Matson PA, Naylor R, Polasky S (2002). Agricultural sustainability and intensive production practices. Nature.

[8] Foley JA (2011). Solutions for a cultivated planet. Nature.

[9] Chen X (2014). Producing more grain with lower environmental costs. Nature.

[10] Zhang X (2015). Managing nitrogen for sustainable development. Nature.

[11] Haegele JW, Cook KA, Nichols DM, Below FE (2013). Changes in nitrogen use traits associated with genetic improvement for grain yield of maize hybrids released in different decades. Crop Sci..

[12] Chen, K. & Vyn, T. J. Post-silking factor consequences for N efficiency changes over 38 years of commercial maize hybrids. *Front. Plant Sci*. 1737, 10.3389/fpls.2017.01737 (2017). 10.3389/fpls.2017.01737 PMC5641558 29075274

[13] DeBruin, J. L., Schussler, J. R., Mo, H. & Cooper, M. Grain yield and nitrogen accumulation in maize hybrids released during 1934 to 2013 in the US Midwest. *Crop Sci*. **57**, 1–16 (2017).

[14] Duvick DN (2005). The contribution of breeding to yield advances in maize (*Zea mays L*.). Adv. Agron..

[15] USDA Foreign Agriculture Service (FAS), https://apps.fas.usda.gov/psdonline/app/index.html#/app/advQuery. Accessed on 18 June 2018 (2018).

[16] Conant RT, Berdanier AB, Grace PR (2013). Patterns and trends in nitrogen use and nitrogen recovery efficiency in world agriculture. Global Biogeochem. Cycles.

[17] Egli DB (2015). Is there a role for sink size in understanding maize population-yield relationships?. Crop Sci..

[18] York LM, Galindo-Castañeda T, Schussler JR, Lynch JP (2015). Evolution of US maize (*Zea mays* L.) root architectural and anatomical phenes over the past 100 years corresponds to increased tolerance of nitrogen stress. J. Exp. Bot..

[19] Campos H (2006). Changes in drought tolerance in maize associated with fifty years of breeding for yield in the U.S. Corn Belt. Maydica.

[20] Reyes A (2015). Soil water capture trends over 50 years of single-cross maize (*Zea mays L*.) breeding in the US corn-belt. J. Exp. Bot..

[21] Tollenaar M, Lee EA (2002). Yield potential, yield stability and stress tolerance in maize. F. Crop. Res..

[22] Moll RH, Kamprath EJ, Jackson WA (1982). Analysis and interpretation of factors which contribute to efficiency of nitrogen utilization. Agron J..

[23] Ciampitti IA, Vyn TJ (2012). Physiological perspectives of changes over time in maize yield dependency on nitrogen uptake and associated nitrogen efficiencies: A review. F. Crop. Res..

[24] Mueller SM, Vyn TJ (2016). Maize plant resilience to N stress and post-silking N capacity changes over time: A review. Front. Plant Sci..

[25] Woli KP, Sawyer JE, Boyer MJ, Abendroth LJ, Elmore RW (2017). Corn era hybrid dry matter and macronutrient accumulation across development stages. Agron. J..

[26] Woli KP (2016). Corn era hybrid response to nitrogen fertilization. Agron J..

[27] Mueller SM, Vyn TJ (2018). Physiological constraints to realizing maize grain yield recovery with silking-stage nitrogen fertilizer applications. F. Crop. Res..

[28] Duvick DN, Smith JSC, Cooper M (2004). Long-term selection in a commercial hybrid maize breeding program. Plant Breed. Rev..

[29] Duvick DN (2005). Genetic progress in yield of United States maize (*Zea mays L*.). Maydica.

[30] Li Y (2011). Increasing maize productivity in China by planting hybrids with germplasm that responds favorably to higher planting densities. Crop Sci..

[31] Hernández F, Amelong A, Borrás L (2014). Genotypic differences among Argentinean maize hybrids in yield response to stand density. Agron. J..

[32] Wang T (2011). Changes in yield and yield components of single-cross maize hybrids released in China between 1964 and 2001. Crop Sci..

[33] Chen K, Camberato JJ, Vyn TJ (2017). Maize grain yield and kernel component relationships to morphophysiological traits in commercial hybrids separated by four decades. Crop Sci..

[34] Di Matteo JA, Ferreyra JM, Cerrudo AA, Echarte L, Andrade FH (2016). Yield potential and yield stability of Argentine maize hybrids over 45 years of breeding. F. Crop. Res..

[35] SAS Institute. Base SAS 9.3 procedures guide. SAS Inst., Cary, NC (2011).

[36] Carmer SG, Walker WM, Self RD (1969). Practical suggestions on pooling variances for F tests of treatment effects. Agron J..

[37] R Development Core Team. R: A language and environment for statistical computing. R Foundation for Statistical Computing, Vienna, Austria (2014).

[38] Gan S, Amasino RM (1997). Making sense of senescence: Molecular genetic regulation and manipulation of leaf senescence. Plant Physiol..

[39] Kosgey JR, Moot DJ, Fletcher AL, McKenzie BA (2013). Dry matter accumulation and post-silking N economy of ‘stay-green’ maize (*Zea mays* L.) hybrids. Eur. J. Agron..

[40] Chen K, Kumudini SV, Tollenaar M, Vyn TJ (2015). Plant biomass and nitrogen partitioning changes between silking and maturity in newer versus older maize hybrids. F. Crop. Res..

[41] Below FE, Christensen LE, Reed AJ, Hageman RH (1981). Availability of reduced N and carbohydrates for ear development of maize. Plant Physiol..

[42] Crawford TW, Rendig VV, Broadbent FE (1982). Sources, fluxes, and sinks of nitrogen during early reproductive growth of maize (*Zea mays L*.). Plant Physiol..

[43] Chen Y (2015). Effects of nitrogen application rate on grain yield and grain nitrogen concentration in two maize hybrids with contrasting nitrogen remobilization efficiency. Eur. J. Agron..

